# Who Cares What the Doctor Feels: The Responsibility of Health Politics for Burnout in the Pandemic

**DOI:** 10.3390/healthcare9111550

**Published:** 2021-11-15

**Authors:** Jasna Karacic, Harold J. Bursztajn, Marianna Arvanitakis

**Affiliations:** 1 International Council of The Patient Ombudsman, 1000 Bruxelles, Belgium; 2 University of Zagreb, 10000 Zagreb, Croatia; 3 BIDMC Psychiatry of Harvard Medical School, Boston, MA 02215, USA; harold_bursztajn@hms.harvard.edu; 4 Department of Gastroenterology, Erasme University Hospital, Université Libre de Bruxelles, 1000 Bruxelles, Belgium; marianna.arvanitaki@erasme.ulb.ac.be

**Keywords:** health policy and politics, burnout and suicide in health-care, moral politics, health systems, COVID-19

## Abstract

Modern health has become a defining facet of contemporary life managed by health policy. The COVID-19 pandemic has significantly affected mental health, resulting in stress and anxiety in doctors’ professional and private life. Since the beginning of the pandemic, doctors have been facing chronic stress, which was reported to the hospital managers and health-care agencies, but nothing was done in the practice to protect them. Although doctors are trained to stay emotionally restrained, a large number of patients in intensive care, along with the personal concerns for their families, has led to burnout. This article highlights the need for health politics to take responsibility for dealing with burnout in health-care workers with a new approach that should help doctors recognize, understand, and manage work-related stress with additional support in the pandemic.

## 1. Introduction

Medicine, unlike politics, is emotionally intertwined [1]. Doctors, acting as professionals, are assumed to be more rational than emotional, but emotions in medicine can still sometimes overwhelm rationality [2]. While all patients want high-quality medical care, at the same time they want empathic doctors who listen to them and understand their needs, feelings, and concerns. Modern health, then, has become a defining facet of contemporary life and its politics all the more vital [3].

The COVID-19 pandemic has challenged many people’s psychological well-being. Along with the mental-health impact of confinement and isolation on the population in general, the mental-health impact on health professionals has become increasingly apparent [4]. Whereas stress, anxiety, and depression-related disorders may be regarded as normal emotional reactions to a pandemic, the causes, incidence, and effects of burnout among health workers need to be addressed.

Although no health worker was spared of burnout, we focused on the doctors in the clinical settings because of their frequent role as head of the health-care team and commander of a considerable clinical resource that requires greater attention is paid to management and leadership skills regardless of specialism [5].

Doctors and hospitals have striven to provide the best health-care for patients during the COVID-19 pandemic [6]. However, after long shifts in hospitals, doctors have reported to health-care agencies, public officials, and the media that they were unable to implement safe policies and practices. Without proper protective equipment, doctors are practically defenseless against COVID-19. Many doctors would have preferred to avoid involvement in politics altogether, but that is no longer possible in the pandemic. Burnout is directly related to political decisions, organization, work structure, and the ability to face up to and deal with stressful factors at work [7].

A challenging problem that arises in this domain is how to protect doctors from burnout and the responsibility of politics for the successful functioning of physicians in delivering care to patients.

## 2. Key Points Disclosure

### 2.1. Is Health Politics Responsible for the Burnout and Suicide of Health Workers in the COVID-19 Pandemic?

The extraordinary stress COVID-19 has placed on doctors led to growing appeals for their protection, yet little attention was paid, and nothing was done in practice [8]. Health-care workers showed signs of chronic stress such as anger, hostility, sadness, or withdrawal. Yet, doctors are taught to remain detached from these emotions in order to maintain the objectivity thought to be crucial to accurate clinical decision making [9].

They have faced challenges in providing invasive procedures to the patients with a decline in mental health and increase in stress levels due to combined work pressure and the fear of infection.

A recent study by Jafree et al. reported alarming signals in disparity training for prevention of COVID-19, no designated trainers, lack of updated medical knowledge about testing and subsequent need for precautions, short supply and quality of surgical masks and gloves, inability to maintain physical distance from patients and co-workers, lack of response from and discriminatory behavior by the administration, lack of time for complete disinfection between emergency cases and inability to test emergency patients [10].

We should call to mind the fact that political leaders were also overwhelmed during the peak of the pandemic, which caused delays in the procedural guidance.

Therefore, while fully acknowledging when health authorities were trying to cope with all the practical emergencies of the days, it was hard to prevent the abovementioned problems. Consequently, some of the health ministers quit over the COVID-19 crisis overwork in particular, because they have largely failed to deliver on the expectations of doctors working in the hospitals.

This is especially the case in view of the fact that the International Council of the Patient Ombudsman in 2020 has received more complaints from the doctors working in clinical settings than the patient’s complaints about the treatments, which raised significant concerns. Doctors complained about the lack of involvement in governance matters and their inability to contribute as team members at the hospital, while patients complained about reducing access to health-care in the hospital lockdown. These patients’ frustrations were often directed at the doctors who care for them, rather than at health policy.

Efforts to give patients the best possible care in the face of the pandemic caused exhaustion followed by burnout syndrome, especially when non-intensive care doctors were trained to assist intensive care specialists in caring for critically ill COVID-19 patients [11]. These problems have immobilized a substantial number of doctors who have become unable to work, which in extreme cases has led to suicide. Hospital managers were not encouraged to explore suicidal ideation, with a range of contemplations, and even early models of stress, whether in or outside the workplace, were unable to capture the human complexity of stress responses [12]. In particular, they failed to incorporate cognitive elements that mediate the perception of stresses and subsequent responses to them. Contradictory ideas and explanations regarding responsibility for suicide also circulate within the health-care sector, which has largely avoided confronting the problem [13].

To overcome this problem, in the next section we demonstrate:

### 2.2. What Is of Global Importance: Rethinking Organization of Health-Care Delivery in the COVID-19 Pandemic with Personal Strategies

Leaders in health politics must, therefore, be aware of the risk to clinicians’ mental health, take care to ensure appropriate working conditions for health-care professionals, and offer national support for facing the challenges generated by the COVID-19 pandemic [14]. 

With “burnout” officially recognized by the World Health Organization (WHO), the responsibility for managing it has shifted away from the individual and towards the organization [15]. It, therefore, becomes part of the mission of health politics to see that health-care organizations take responsibility for burnout prevention. All affected agencies must work to understand and address burnout syndrome both preventively and remedially [16]. It would be especially important to have a consensus between the Center for Disease Control and Prevention (CDC) and the WHO as the main health authorities.

It has been almost two years waiting to release a joint statement on WHO estimates of health-care workers deaths due to COVID-19 that call for concrete action to better protect health-care workers worldwide from COVID-19. The statement also urges political leaders and policymakers to do all within their power to make regulatory, policy, and investment decisions that ensure the protection of health-care workers [17].

Furthermore, the general conference of the International Labour Organization, having received the proposal made by the Conference Committee on the Response to COVID-19, considers the urgent need for a global call to action to ensure a human-centered recovery from the COVID-19 crisis that is inclusive, sustainable and resilient.

Protection from this resolution provides that workers at higher risk of exposure to COVID–19 and those at greater risk of negative health impacts, such as health-care workers and all other frontline workers, including those working transnationally, have access to vaccines, personal protective equipment, training, testing, and psychosocial support, and that they are adequately remunerated and protected at work, including against excessive workloads [18]. Accordingly, more guidelines also need to be developed and communicated to medical teams about treatment options for minimally invasive procedures versus complex interventions and the related risks of transmission regarding doctors’ own safety [19]. However, the provision protocols were often taken on an ad hoc basis, were reactive to the circumstances, and lacked an anticipatory overall hospital management system.

Therefore, now WHO is working on guidelines for prevention strategies for health-care organizations, most of which still have no idea what to do about burnout [20]. In accepting responsibility for dealing with this very real complication of the pandemic, health-care organizations need to develop and implement recommendations that are sensitive to the mental health needs of health-care workers. 

The psychological support program could help consistent effectiveness on mental health and quality of life as well as on productivity in health-care settings.

**Figure 1:**
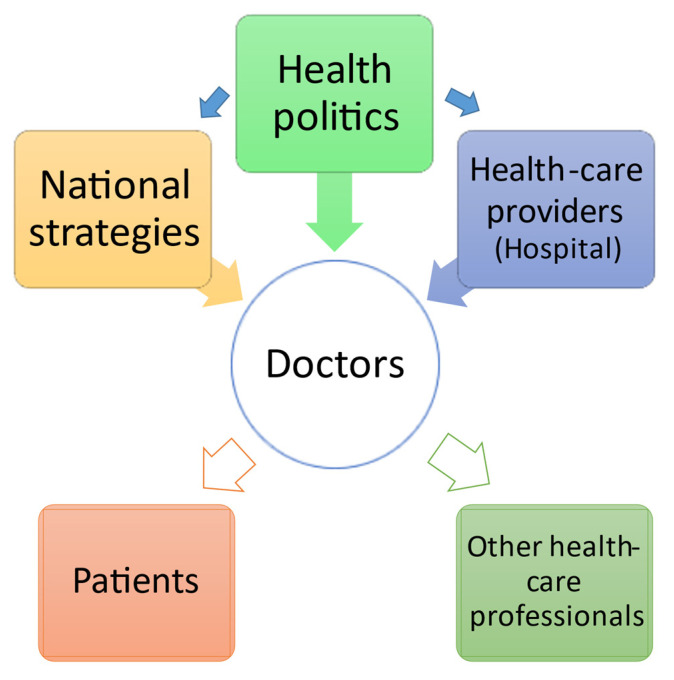
The organizational structure of communicating responsibility in health-care.

In grief-stricken times the doctors as leaders of the health-care team in providing care to the patients, to remain well in revitalizing hospital work into a post-COVID-19 world should receive additional support from health politics and possibilities to address all questions and concerns in their work. Organizational politics should inform and instruct hospital management, and hospital leaders could then implement protocols to protect their health workers (Figure 1).

For instance, we need to create the time for clinicians to grieve. This may include time to speak to suffering patients and to concerned and grieving families; time to grieve for patients lost and for one’s own at times helplessness and ever-present vulnerability [21].

The additional burden during this period is also personal health or family issues from the doctors’ side related (or not) to COVID. We consider our doctors to be superheroes, but they are not. They can also be concerned by health problems for their elderly parents, their siblings, and children, which confirms that extra support and compassion are needed during these times. The message within the image that doctors are superheroic, self-sacrificing, and able to patiently and effectively provide care to all those in need, ended up putting to recruit them to the war effort, leaving no space for thinking of themselves.

The significant concern is bringing the virus home to their families. On the one hand, there is a fear that if they fall ill they will be betraying the health system and their patients as they will not be able to contribute to the COVID fight, while on the other hand, as potential virus carriers, there is a fear to have any close interaction with their families, followed by difficulties in the emotional attachment as the inability of hugging kids or parents.

Therefore, to complement the clinical efforts in preventing the spread and treating of COVID-19 cases, available psychometric instruments could be used in assessing and allaying fears of COVID-19, such as the Fear of COVID-19 Scale (FCV-19S) [22].

On a clinical level, providing psychological care to employees generates a higher perceived workplace health support, which in turn positively influences productivity [23].

This allows the conclusion that whatever the differences between health politics and politics in other contexts, health politics has an important role to play in understanding and taking responsibility for coping with a burnout in health-care workers. 

We highlight the importance of developing a consensus definition of burnout and of standardizing measurement tools to assess the effects of chronic occupational stress on doctors engaged in crisis management. 

Mistakes and omissions made by health authorities, in the beginning, must not be repeated. Following all warnings issued so far, there are no more excuses. It is necessary to make sure crisis-relevant countermeasures for burnout and to ensure support for swift decision-making that will steer investment and action in strengthening doctors mental health. The emergency preparedness and response authority worldwide actions should fully involve and consult doctors working directly with the patients. This perspective improves capacities by fostering knowledge and skills through targeted national strategies in together coordination with health politics, hospital managers, and doctors.

A new approach, therefore, is needed to help doctors recognize, understand, and manage work-related stress and burnout. This effort is essential for everybody working in clinical settings. At the same time, health politics should see its role as supporting doctors, not setting itself up as superior to them.

Health-care policy needs to listen to and respect the discourse between clinicians and their patients. What is the best treatment course for an individual patient is a question that can best be answered in the context of the individual clinician–patient relationship.

## Data Availability

Not applicable.

## References

[1] Bambra C., Smith K.E., Pearce J. (2019). Scaling up: The politics of health and place. Soc. Sci. Med..

[2] Kerasidou A., Horn R. (2016). Making space for empathy: Supporting doctors in the emotional labor of clinical care. BMC Med. Ethics..

[3] Moudatsou M., Stavropoulou A., Philalithis A., Koukouli S. (2020). The role of empathy in health and social care professionals. Healthcare.

[4] Shah K., Chaudhari G., Kamrai D., Lail A., Patel R.S. (2020). How Essential Is to Focus on Physician’s Health and Burnout in Coronavirus (COVID-19) Pandemic?. Cureus.

[5] Wilkie V. (2012). Leadership and management for all doctors. Br. J. Gen. Pract..

[6] Nyquist J.G. (2014). What doctors feel: How emotions affect the practice of medicine. J. Chiropr. Educ..

[7] Choflet A., Packard T., Stashower K. (2021). Rethinking organizational change in the COVID-19 era. J. Hosp. Manag. Health Policy.

[8] Angoff N.R. (2002). Making a Place for Emotions in Medicine. Yale J. Health Pol’y. L. Ethics.

[9] Maslach C., Leiter M.P., Jackson S.E. (2012). Making a significant difference with burnout interventions: Researcher and practitioner collaboration. J. Organ. Behav..

[10] Jafree S.R., Momina A.U., Malik N., Naqi S.A., Fischer F. (2021). Challenges in providing surgical procedures during the COVID-19 pandemic: Qualitative study among Operating Department Practitioners in Pakistan. Sci. Prog..

[11] Engberg M., Bonde J., Sigurdsson S.T., Møller K., Nayahangan L.J., Berntsen M., Eschen C.T., Haase N., Bache S., Konge L. (2021). Training non-intensivist doctors to work with COVID-19 patients in intensive care units. Acta Anaesthesiol. Scand..

[12] Harmer B., Lee S., Duong T.V.H., Saadabadi A. (2021). StatPearls [Internet]. Suicidal Ideation.

[13] Carpenter D. (2012). Is Health Politics Different?. Annu. Rev. Political Sci..

[14] Silva-Gomes R.N., Silva-Gomes V.T. (2021). COVID-19 pandemic: Burnout syndrome in healthcare professionals working in field hospitals in Brazil. Enferm. Clin..

[15] World Health Organization Burn-out an “Occupational Phenomenon”: International Classification of Diseases. https://www.who.int/news/item/2805-2019-burn-out-an-occupational-phenomenon-international-classification-of-diseases.

[16] Stone K.W., Kintziger K.W., Jagger M.A., Horney J.A. (2021). Public Health Workforce Burnout in the COVID-19 Response in the U.S. Int. J. Environ. Res. Public Health.

[17] (2021). The Impact of COVID-19 on Health and Care Workers: A Closer Look at Deaths.

[18] International Labour Organization. Global call to action for a human-centred recovery from the COVID-19 crisis. Proceedings of the Texts adopted-Resolution. International Labour Conference.

[19] Sarker S.K., Vincent C. (2005). Errors in surgery. Int. J. Surg..

[20] Moss J. (2021). Beyond burned out. Harvard Business Review.

[21] Bursztajn H.J., Feinbloom R.I., Hamm R.M., Brodsky A. (1981). Medical Choices, Medical Chances: How Patients, Families, and Physicians Can Cope With Uncertainty.

[22] Ahorsu D.K., Lin C.Y., Imani V., Saffari M., Griffiths M.D., Pakpour A.H. (2020). The Fear of COVID-19 Scale: Development and Initial Validation. Int. J. Ment. Health Addict..

[23] Dalmasso G., Di Prinzio R.R., Gilardi F., De Falco F., Vinci M.R., Camisa V., Santoro A., Casasanta D., Raponi M., Giorgi G. (2021). Effectiveness of Psychological Support to Healthcare Workers by the Occupational Health Service: A Pilot Experience. Healthcare.

